# Challenges in supporting lay carers of patients at the end of life: results from focus group discussions with primary healthcare providers

**DOI:** 10.1186/s12875-018-0816-4

**Published:** 2018-07-13

**Authors:** Katja Krug, René Alexander Ballhausen, Regine Bölter, Peter Engeser, Michel Wensing, Joachim Szecsenyi, Frank Peters-Klimm

**Affiliations:** Department of General Practice and Health Services Research, University Hospital Heidelberg, Im Neuenheimer Feld 130.3, 69120 Heidelberg, Germany

**Keywords:** Family caregivers, Focus group discussions, Primary care, Healthcare provider in general practice, End-of-life care, Palliative care

## Abstract

**Background:**

Family caregivers (FCGs) of patients at the end of life (EoL) cared for at home receive support from professional and non-professional care providers. Healthcare providers in general practice play an important role as they coordinate care and establish contacts between the parties concerned. To identify potential intervention targets, this study deals with the challenges healthcare providers in general practice face in EoL care situations including patients, caregivers and networks.

**Methods:**

Focus group discussions with general practice teams in Germany were conducted to identify barriers to and enablers of an optimal support for family caregivers. Focus group discussions were analysed using content analysis.

**Results:**

Nineteen providers from 11 general practices took part in 4 focus group discussions. Participants identified challenges in communication with patients, caregivers and within the professional network. Communication with patients and caregivers focused on non-verbal messages, communicating at an appropriate time and perceiving patient and caregiver as a unit of care. Practice teams perceive themselves as an important part of the healthcare network, but also report difficulties in communication and cooperation with other healthcare providers.

**Conclusion:**

Healthcare providers in general practice identified relational challenges in daily primary palliative care with potential implications for EoL care. Communication and collaboration with patients, caregivers and among healthcare providers give opportunities for improving palliative care with a focus on the patient-caregiver dyad. It is insufficient to demand a (professional) support network; existing structures need to be recognized and included into the care.

## Background

Most patients at the end of life (EoL) are at home and receive out-patient care in Germany. They face the challenge to organise their lives and optimize their quality of life [1]. Decisions on the place of EoL care are influenced by available social support networks (involving lay carers such as family members, friends, and neighbours), preferences of patients, and supply of professional healthcare providers [2]. Patients, whose families are involved in their care as family caregivers and whose families are able to cope with the palliative care situation, are less likely to receive hospital care at the EoL [3] and more likely to die at home [4]. Admission to hospitals during the last days of life [5, 6] are often due to family caregivers feeling overburdened and without adequate professional support [7].

Lay carers are mostly family caregivers, friends and neighbours. Spouses comprise the largest part of those who support and care (28%), followed by daughters (26%) and parents (14%) [8]. These caregivers often devote a large part of their time (on average 37 h per week) to the care of a single patient [8] which leads to social isolation and higher perceived caregiver burden [9]. Caregivers are also prone to physical impairments, i.e. sleep deprivation and weight loss [9]. Caregiving is not only physically demanding, lay carers often develop psychological symptoms, i.e. depression and anxiety [9, 10], they experience stress due to caregiving [11] and may need support themselves. A higher caregiver burden is associated with lower education and living together with the care recipient [9]. Lay carers may receive support from other lay carers as well as professional healthcare providers. Coordination of care across all care givers is regarded a high priority by patients [12].

In Germany, palliative care has been developed as part of the hospice movement, which cumulated in the foundation of the first palliative care unit in 1983 [13]. For outpatients with life-limiting advanced diseases and complex care needs, a national bill on specialized ambulatory palliative care (SAPV, §37b social security statutes book V) was introduced in 2007. Approximately 10% of outpatients were estimated to be in need of SAPV [14]. For palliative outpatients without complex care needs, general practitioners (GPs) are traditionally in the best position to provide palliative care, including EOL care. A specific qualification program for GPs in palliative care or structured palliative care, comparable to the Gold Standards Framework in the United Kingdom [15], does not exist nationwide. There are local initiatives available, i.e. a palliative care initiative in northern Baden (PAMINO [16]). They also provide a platform for networking among general practices.

Networks can be used to share experiences with colleagues, since in Germany, general practices are mainly single-handed with few staff (medical assistants) [17]. Medical assistants take over administrative tasks from physicians and are also increasingly involved in patient care, i.e. disease and care management and home visits [17]. The practice teams should not only take care of the medical problems of patients, but also give social and logistic support. With patients at the EoL, healthcare providers in general practice are confronted with a complex situation involving both patients and caregivers as well as other healthcare providers. This study aims to close the knowledge gap about the challenges healthcare providers in general practice recognize which are posed by patients, lay carers, and professional carers in the care situation at the EoL of a patient and how the providers deal with those challenges.

## Methods

The study was part of the PalliPA project (Verbesserung der häuslichen Versorgung von Palliativpatienten durch Unterstützung pflegender Angehöriger – Improvement of palliative care at home by supporting family caregivers) and focused on one aspect of EoL care important throughout the care process: the role of general practice teams in supporting family caregivers of patients at the EoL [18]. The project was framed as an exploratory trial for a subsequent implementation study. Before an intervention was developed, implemented and evaluated, a concurrent observation of the care problem was necessary. A qualitative approach with focus group discussions was used to gather a wide range of ideas and opinions regarding challenges in EoL care situations from healthcare providers in general practice. Interviews allow participants to discuss a given topic from their personal point of view, whereas in focus group discussions participants can discuss among themselves which can result in an additional source of information [19]. The focus group discussions in this study were structured with a questioning guideline covering aspects of good palliative care, identification and support of lay carers (Table 1). The questioning guideline was pilot tested with 2 palliative care physicians before conducting the formal focus group discussions.

**Table 1:** Questioning guideline (main questions for reported analysis)

What motivated you at first to engage in palliative medicine?	
What facilitates or hampers successful palliative care from your point of view?	
Thinking of your last palliative care patient:	
When did the family caregivers (FCs) get involved?	
What was triggering the approach to the FCs?	
Who initiates the contact between practice and FCs?	
In which situations do you contact FCs directly?	
What role does the staff actually play regarding the contact to the FCs?	
How do you know if FCs are burdened?	
What do you as a practice team do to support FCs?	
What offers of assistance do you give?	
How do FCs react to offers of assistance?	
Can you remember a situation where FCs were especially reluctant or demanding? How did you react?	
Do you talk about the possibility to admit the patient to a hospital? In which situations?	
What has to change to enable your practice to better support FCs?	
What kind of support would you like to have?	

To take part in the focus group discussions, healthcare providers in general practice with a special interest in palliative care were approached. This was based on the premise that those practice team members would be more highly motivated to identify and develop a gold standard for palliative care in German general practice than a representative sample. This approach follows purposeful sampling with the aim of a homogeneous sample [20]. The approached recruitment sample consisted of healthcare providers in general practice from the federal state of Baden-Wuerttemberg. It was intended that participants were currently taking care of palliative patients. Contact information for potential participants was sourced from the publicly available register of the Association of Statutory Health Insurance Physicians (Kassenärztliche Vereinigung) Baden-Wuerttemberg. All general practitioners with an additional qualification in palliative medicine were identified and invited to participate in the study. Invitations were mailed to 258 general practices with 275 general practitioners.

Four 90-min focus group discussions were part of 2 recruitment meetings where the study was introduced and some background information on lay carers provided (i.e. facts on how many people take care of a family member underlining the importance of the topic). For the purpose of getting a multi-faceted picture, focus group discussions were mixed (general practitioners (GPs) and medical assistants), but GP and assistants from one practice did not attend the same focus group discussion. The focus group discussions were moderated by one of 3 members of the research team, who all had extensive experience in conducting focus group discussions and had also been involved in developing the questioning guideline (2 medical doctors (PE, FPK) experienced in qualitative research, 1 social scientist (KK)); the moderating medical doctors were also working as GPs and had an additional qualification in palliative medicine. The focus group discussions took place in the Department of General Practice and Health Services Research; GPs interested in the study but not able to attend the focus group discussions were asked to take part in a telephone interview covering the same aspects as the focus group discussions. Before conducting focus group discussions and interviews, all participants provided written informed consent to participating in, audio-recording and analysis of the focus group discussions.

The structured focus group discussions and interviews were audio-recorded and transcribed verbatim. Transcripts were analysed with content analysis [21] using ATLAS.ti. Two researchers (RAB, KK) reviewed and coded the transcripts independently. First, codes were derived deductively on the basis of the questioning guideline (Table 1); additional topics were derived inductively from the transcripts. During the iterative coding process, the codes were regularly compared, discussed and adapted for further use in the analysis. Finally, consensus was reached with a third researcher (FPK). The whole research team discussed and interpreted the results. While the analysis was performed on the original German data, quotes are translated into English for reporting in this paper.

## Results

Thirty-five practices responded to the invitation (14%), of which 22 declined participation. The most common reason given for not taking part was lack of time. Four focus group discussions were conducted with 19 participants from 11 practices (12 GPs, 7 medical assistants), two additional telephone interviews with GPs who were not able to participate in the focus group discussions. Participant characteristics are given in Table 2. Data saturation was reached after 3 focus group discussions; no new topics emerged in the last focus group discussion and in the telephone interviews.

**Table 2:** Description of participants

	Abbreviation	Details	Professional experience^a^
Focus group discussion 1	S1	practice of GP3	additional palliative care certificate since 2 years
	S2	practice of GP4	
	S3	practice of GP4	since 10 years in the practice
	GP1	with S5	since 22 years in the practice, qualification in palliative medicine since 1 year
	GP2	with S4	since 26 years in the practice, qualification in palliative medicine since 5 years, SAPV
Focus group discussion 2	S4	practice of GP2	since 9 years in the practice, additional palliative care certificate
	S5	practice of GP1	
	GP3	with S1	since 24 years in the practice, qualification in palliative medicine since 1 year
	GP4	with S2 and S3	general practitioner since 10 years, since 6 years in the practice
	GP5		since 27 years in the practice
Focus group discussion 3	GP6	with S7	since 13 years in the practice, qualification in palliative medicine since 7 years
	GP7	with S6	since 12 years in the practice, qualification in palliative medicine since 3 years, SAPV
	GP8		since 1 year in practice after neurosurgery for 14 years and general practice for 4 years
	GP9	same practice as GP11	qualification in palliative medicine since 2 years
Focus group discussion 4	S6	practice of GP7	since 1 year in the practice after completing training in another practice
	S7	practice of GP6	since 5 years in the practice after working in hospital
	GP10		since 10 years in the practice, qualification in palliative medicine since 5 years, care of palliative patients for at least 20 years
	GP11	same practice as GP9	qualification in palliative medicine since 3 years
	GP12		since at least 20 years in the practice, qualification in palliative medicine since 1 year
Telephone interview 1	GP13		
Telephone interview 2	GP14		since 3 years in the practice after geriatrics

^a^as deductible from focus group discussions and interviews, *S* Staff, *GP* General practitioner, *SAPV* Member of a specialized ambulatory palliative care team (Spezialisierte Ambulante Palliativversorgung)

**Fig. 1:**
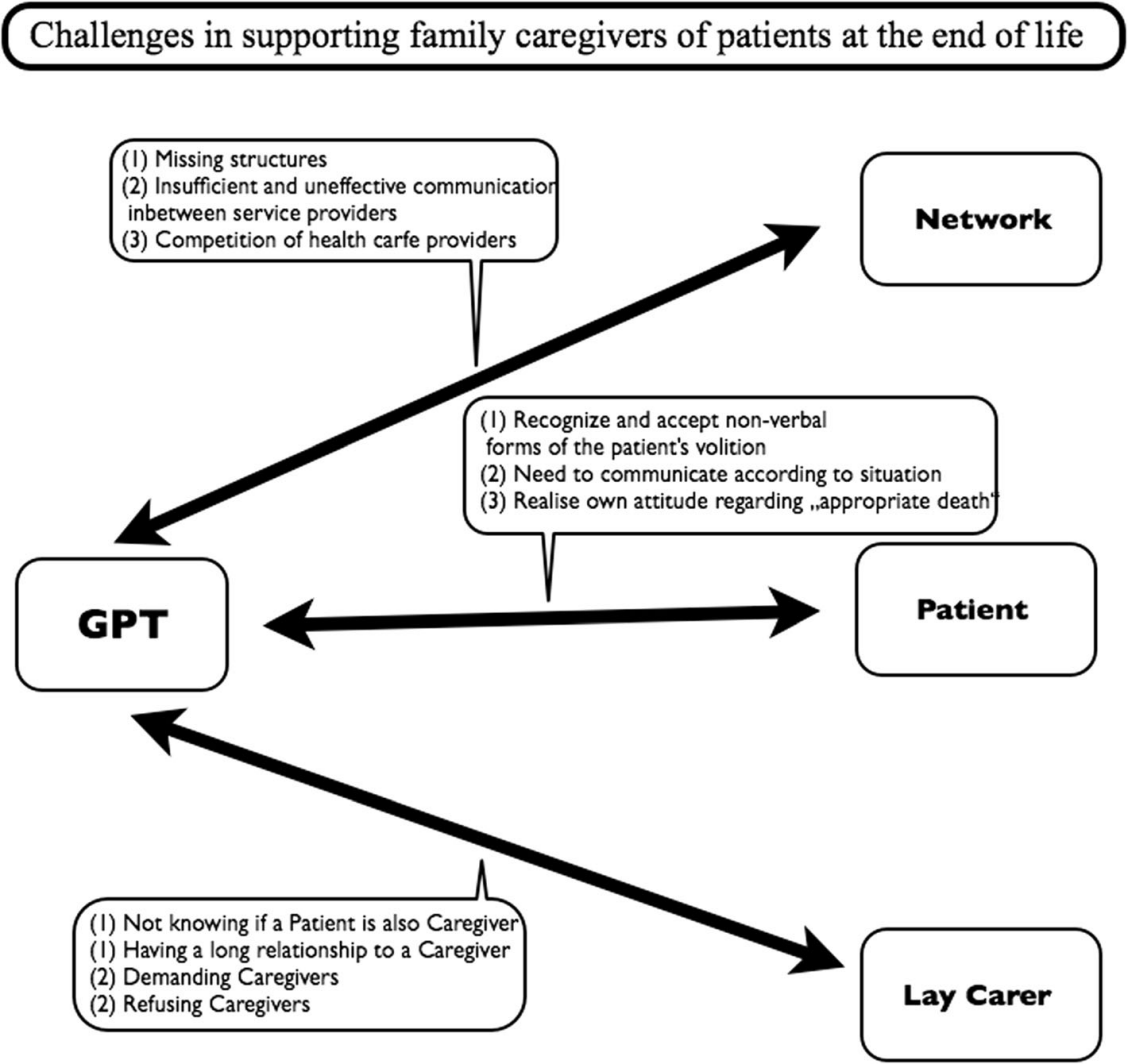
Overview of challenges reported by members of general practice teams (GPT)

In the perspective of the participants from general practices, 3 main categories with 8 subcategories emerged: challenges with the patients, challenges with the caregivers, and challenges within the professional network. An overview is given in Fig. 1.

### Challenges with patients

The majority of the participants faced challenges in dealing with patients, especially when they encountered resistance or reactions they did not anticipate: (1) they needed to recognize and accept non-verbal forms of the patient’s volition, (2) they needed to communicate according to the situation, and (3) they needed to realize their own attitudes regarding an “appropriate” death.

(1) Healthcare providers recognized patients who were not able to communicate verbally as a group where palliative care has to be accomplished in the best interest of the patient. Participants particularly stressed the point of interpreting patient’s behaviour:

*With dementia patients […] if someone does not eat or drink anymore, that’s also a form of volition. Then I would absolutely refrain from any infusion or PEG (percutaneous endoscopic gastrostomy tube) or subcutaneous or else. (GP3).*

(2) Most of the practice team members acknowledged the need to prepare patients for the time to come and to organize support on time, but they also experienced patients declining help if they did not perceive it as urgent:

*I sometimes notice that if you want to organise things ahead […] but the patient is reasonably well, they decline. (GP6).*

(3) The majority of participants did not explicitly formulate their attitudes towards an “appropriate” death, but described how their own opinion influenced care decisions:

*For 2 years, I had an old lady, where I asked the relatives to get her into a nursing home, since the couple at home were both heavy alcoholics. And I thought it would be actually awful for this old lady if she had to die at home. (GP12).*

### Challenges with lay carers

In their contact with caregivers, participants reported a wide range of relations to caregivers: (1) not knowing the relatives of a patient, not knowing if a patient is also a caregiver, but also having a long and close relationship to the caregiver. Healthcare providers were also (2) challenged by either demanding or refusing family caregivers and found individual ways to cope in such situations.

(1) In Germany, there is no obligation to register with a GP (practice). Patients may freely choose their GP. Unless GPs document explicitly the social case history or patients report for themselves, GPs and their practice teams do not necessarily know if a patient has lay carers or is a caregiver to a family member themselves. If more than just one family member are patients in the practice, the social structures of the patients were better known to the healthcare providers:

*We definitely have people, where we don’t know what’s happening at home. (GP7).*

*Usually, they are patients we’ve known for years. We’ve known them since we’ve had the practice. And then we know the family structure, too. (GP9).*

(2) Demands of family caregivers focused either on the care for the patient or on the caregivers themselves. For healthcare providers in general practice the request for support often came too late or was considered inappropriate:

*Of course it happens that the relatives come in a state of absolute exhaustion and then dump everything on the table and say: Now it doesn’t work anymore. And now you (the doctor) best do something immediately. (GP4).*

Participants also described family caregivers, who were not responsible enough or taking sufficient action themselves from a practice’ point of view:

*Caregivers, who expect, that others …, that […] everything will be done, and who need to be nudged to become active by themselves. (GP14).*

In some cases, demands of caregivers on patients were also brought up, especially when it came to decisions about further therapies closer to the EoL:

*It goes both ways. There are relatives, who [struggle to accept the patient does not] want any therapy anymore, and then try to really convince the patient to do otherwise. (GP6).*

Participants reported that caregivers (and patients) refused support sometimes rigorously, especially if they had the feeling of being able to cope:

*A nursing home was not an option for them both. They didn’t want to. The doctor advised to go to a nursing home. No. (S5).*

Healthcare providers in general practice tried to give support at an early stage and to emphasize the caregiver’s health. They also offered help for care aspects that were easier to accept for the family caregivers:

*If you offer something in this (early) stage, it will be mostly rejected. [They say:] “No, I don’t need that yet or it is okay or I can do it.” […] would be ideal, to get there at the right time. (GP4).*

The participants perceived family caregivers as important supporters in the care of the patients. As such, they saw the necessity of treating patients and caregivers as a unit, with the caregivers’ health influencing the trajectory of the patient’s disease:

*I try to address diplomatically that they are the most important persons and that their health is enormously important for the patient. Even if the caregiver is not one of my patients, it is the interest of the patient to have a healthy caregiver in all respects. Mentally, and physically. (…) That the quality of the care deteriorates if the exhaustion gets too much. (GP4).*

### Challenges within the professional network

To provide high-quality palliative care, healthcare providers in general practice need to work together with local professional support. They are involved in a multidisciplinary network which facilitates cooperation and involves nursing services, physiotherapy, spiritual care, volunteers and others.

At the same time, practice team members are faced with challenges within the network: (1) structures are missing, (2) the communication between cooperating partners does not involve all relevant information, and (3) partners who should cooperate in patient care are also competitors on the healthcare market.

(1) Although there is a widespread professional network, not all services are locally available. Especially inpatient structures are missing in some areas:

*We don’t have a hospice, we don’t have a palliative care unit. (GP12).*

(2) Relevant information is not shared between service providers on the interface of healthcare facilities; it is not given timely and unstated reasons for omission lead to unrequested interventions:

*This gap between hospital and practice. Discharge on Friday afternoon, should get antibiotics and analgesics etc. I see the fax message maybe Saturday morning or Monday morning. (GP5)*.

*An 88-year-old was discharged. I just had her admitted for transfusion, because she was anaemic again and again, and I almost knew there had to be a malignancy behind that. So I had her specifically admitted only for transfusion, and still they did a colonoscopy or tried to do it. Stenosing colorectal carcinoma, and now the clinic offered her an operation. (GP7)*.

(3) A big challenge perceived by some practice team members was the building of a cooperating network where the potential partners are also competitors for patient care:

*The building of the team was difficult, since the nursing services are firstly competitors and sometimes quarrelling among themselves. But since we physicians took the initiative (…), we brought them all together using really a lot of diplomacy and tact. And so all of a sudden, the structure, the nursing services among themselves, has changed. (GP7)*.

## Discussion

Healthcare providers in general practice are faced with a multitude of challenges in EoL care in communicating with patients, lay carers and other professional healthcare providers. From their point of view, practice team members have to deal with an appropriate communication with patients, interpreting patients’ non-verbal messages and reacting accordingly. They need to identify lay carers and to find ways to deal with inappropriate demands and unnecessary refusals of support. They perceive themselves as an important part of the healthcare network, but also report difficulties in communication and cooperation with other healthcare providers.

To recognize the appropriate time to communicate with patients and to perceive patients’ behaviour as a non-verbal expression of their wishes are essential communication skills in general practice. In EoL care, not only communication with patients but also with caregivers and within the professional network is of paramount importance. General practice teams need to be aware of the high potential of challenging situations as described in our study.

A systematic review showed that direct support of caregivers can reduce their psychological distress [22]. The interventions described comprise home visits by nurses of up to 1.5 h which are not feasible for German general practices. The topics important for family caregivers [23], on the other hand, are addressed in general practice, i.e. caregivers learn about symptom management, their health needs are discussed and they receive support provided by social networks and health services providers [24]. The results of the present study additionally stress the importance of communication with patients, caregivers and other health care providers to bring offers and demands of support together.

Another systematic review [25] summarized facilitators for a good patient-physician communication: the availability of the GP and the initiation of EoL issue discussions. Our participants additionally stressed the point of finding the right moment to initiate discussions with patients and caregivers to lead to effective results. Thus, communicating with and supporting the patient-caregiver *dyad* needs to be individually targeted [26]. Additionally, it underscores the importance of integrating early palliative care also in the primary care of diseases other than cancer [27].

Contrary to the situation in several other countries, such as the UK, general practices in Germany are not contractually required to identify (family) caregivers [28]. Still, the challenges for identification are comparable: family caregivers perceive themselves as relatives in the first place before considering themselves as carers [28]. As our participants reported, family caregivers have to be made aware of their need for support, even though they might refuse it. The healthcare providers perceived patients and caregivers as a unit of care and caregivers as potential “hidden patients” [29] and tried to support them to prevent caregiver collapse.

The perception of patients at the EoL and their caregivers leads to reflections on professional attitude which emerge not only in communication but also in the expression of practice team members’ personal values and the evaluation of cooperation within the professional network.

While working with palliative patients and their family caregivers, healthcare providers in general practice are faced with their own assumptions of a “good” death, which goes further than the biomedical and psychosocial perspective [30] and might not concur with the views of patients and caregivers [31]. In our study, this topic was not explicitly addressed and thus, is not within the scope of this paper. In addition, to further analyse implicit assumptions of healthcare providers, another methodological approach would be needed.

The communication and cooperation with other healthcare providers needs to be optimized. The statements of the participants demonstrate that the communication has to be improved in both ways: from the practice to other service providers as well as from other service providers to the practice. All partners have to clarify their role and their expectations. This might also strengthen the communication with caregivers, who reported both unmet psychosocial needs and poor coordination of health-care services in a systematic review [32].

The results of our study show challenges of German healthcare providers in general practice, which were considered challenges in other European countries as well. In the UK, the Gold Standards Framework [33] addresses those topics and gives practice teams a guideline to deal with i.e. communication, coordination, and carer support. The assessment of burden in family caregivers poses a major challenge for general practitioners. Approaches for assessment have been described by Adelman et al. in a review [9], but to date in Germany no formal assessment strategies have been implemented. However, recent initiatives in the form of “burden relief programs for family caregivers”, e.g. by the national elderly care insurance fund, have been introduced [34]. Nevertheless, awareness of such initiatives among general practitioners needs to be raised. Promotional information could be shared through GP networks. Recent evidence indicates German GPs actively utilize the German palliative care guidelines [35]. Information on burden relief programs for family caregivers could be included in this resource. Certainly there is room for improvement of palliative care in German primary care to be further improved on policymaking, practical and educational level [36]. Furthermore, our results are in agreement with other studies dealing with organizational challenges [37], the importance of communication and continuity in patient care as well as to the family caregivers [38, 39].

### Strengths and weaknesses of the study

This study is part and the first step of a project to design and develop an intervention that may be implemented and evaluated in primary care to support lay carers. Focus group discussions were conducted with healthcare providers from general practices who were potential participants in the project [18]. They aimed at identifying facilitators and barriers of optimal palliative care in general practice, particularly on supporting lay caregivers. As a result of the focus group discussions, interventions were developed together with participants in the further project process [18].

The results are based on focus group discussions with a highly selective sample. We purposefully included primary healthcare providers with a special interest in palliative care. Thus, the participants were probably more familiar with the situation of family caregivers of patients at the EoL and could share the strategies they had developed to deal with the specific challenges occurring in such complex situations. We did not countercheck the impressions of the participating healthcare providers with the views of patients, lay carers, and other professional caregivers. Therefore, their views remain highly subjective and might be due to individual perceptions. In spite of this subjectivity, the topics were generally taken up by the participants of the focus group discussions. Reported experiences of general practice teams from our study are in part addressed in findings of studies examining the patient/caregiver end of the EoL care continuum. In particular, communication difficulties with general practitioners, inconsistencies and discontinuity of care have been reported [40].

Although it is a sample of health care providers, participants could vividly report on the challenges they are faced with in palliative care. However, due to the mixture of GPs and medical assistants in the focus group discussions, statements might be influenced by a possible social status gradient. We sought to minimize this potential bias by assigning members of the same practice team to different focus group discussions that took place in parallel. Additionally, the experienced moderators were conscious to invite reticent participants to share their thoughts and opinions. Nonetheless, there could be other challenges participants were not aware of and therefore did not report. Still, it could be assumed that the challenge for healthcare providers in general practice teams without a special interest in palliative care is even greater.

## Conclusion

Healthcare providers in general practice identified many relational challenges in daily primary palliative care. Based on our findings, strategies to resolve these may concern the dimensions knowledge, professional attitude and skills which could be further supported and developed as part mixed interventions with educational and organisational elements. Our results indicate that it is insufficient to demand a support network; existing structures need to be recognized and included into the care in general practices. At the same time, motivations contrary to collaborative care due to a competitive healthcare market need to be considered. Additionally, healthcare providers in general practice should assess the relationship between patients and caregivers as a significant reason for accepting or denying support.

## Abbreviations

EoL: End of life
FCGs: Family caregivers
GPs: General practitioners
PalliPA: Project Verbesserung der häuslichen Versorgung von Palliativpatienten durch Unterstützung pflegender Angehöriger (Improvement of palliative care at home by supporting family caregivers)
PAMINO: Palliativmedizinische Initiative Nordbaden (palliative care initiative in northern Baden)
PEG: Percutaneous endoscopic gastrostomy tube
SAPV: Spezialisierte ambulante Palliativversorgung (specialized ambulatory palliative care)

## References

[1] Gomes B, Higginson IJ (2006). Factors influencing death at home in terminally ill patients with cancer: systematic review. BMJ.

[2] Murray MA, Fiset V, Young S, Kryworuchko J (2009). Where the dying live: a systematic review of determinants of place of end-of-life cancer care. Oncol Nurs Forum.

[3] Gott M, Frey R, Robinson J, Boyd M, O’Callaghan A, Richards N, Snow B (2013). The nature of, and reasons for, ‘inappropriate’ hospitalisations among patients with palliative care needs: a qualitative exploration of the views of generalist palliative care providers. Palliat Med.

[4] Kern M, Wessel H, Ostgathe E (2007). Ambulante Palliativbetreuung - Einflussfaktoren auf eine stationäre Einweisung am Lebensende. Z Palliativmed.

[5] Jordhoy MS, Fayers P, Saltnes T, Ahlner-Elmqvist M, Jannert M, Kaasa S (2000). A palliative-care intervention and death at home: a cluster randomised trial. Lancet.

[6] Howat A, Veitch C, Cairns W (2007). A retrospective review of place of death of palliative care patients in regional North Queensland. Palliat Med.

[7] Tiernan E, O’Connor M, O’Siorain L, Kearney M (2002). A prospective study of preferred versus actual place of death among patients referred to a palliative care home-care service. Ir Med J.

[8] Döhner H, Kofahl C, Lüdecke D, Mnich E (2007). The National Survey Report for Germany. EUROFAMCARE Services for Supporting Family Carers of Older Dependent People in Europe: Characteristics, Coverage and Usage.

[9] Adelman RD, Tmanova LL, Delgado D, Dion S, Lachs MS (2014). Caregiver burden: a clinical review. JAMA.

[10] Lavela SL, Ather N (2010). Psychological health in older adult spousal caregivers of older adults. Chronic Illn.

[11] Lund L, Ross L, Petersen MA, Groenvold M (2014). Cancer caregiving tasks and consequences and their associations with caregiver status and the caregiver's relationship to the patient: a survey. BMC Cancer.

[12] Han PK, Rayson D (2010). The coordination of primary and oncology specialty care at the end of life. J Natl Cancer Inst Monogr.

[13] Cremer-Schaeffer P, Radbruch L (2012). Palliative care in the light of legal and regulatory requirements in Germany. Bundesgesundheitsblatt Gesundheitsforschung Gesundheitsschutz.

[14] Voltz R (2008). Palliativmedizin: Eine Disziplin für den “ganzen Menschen”. Dtsch Arztebl.

[15] King N, Thomas K, Martin N, Bell D, Farrell S (2005). Now nobody falls through the net’: practitioners’ perspectives on the gold standards framework for community palliative care. Palliat Med.

[16] Engeser P, Reininghaus W, Zeise-Suess D, Wiesemann A (2003). Palliativmedizin in Nordbaden (PAMINO-Projekt) Welche Einstellungen haben nordbadische Hausärzte zur Palliativmedizin. Z Allg Med.

[17] Freund T, Everett C, Griffiths P, Hudon C, Naccarella L, Laurant M (2015). Skill mix, roles and remuneration in the primary care workforce: who are the healthcare professionals in the primary care teams across the world?. Int J Nurs Stud.

[18] Hermann K, Boelter R, Engeser P, Szecsenyi J, Campbell SM, Peters-Klimm F (2012). PalliPA: how can general practices support caregivers of patients at their end of life in a home-care setting? A study protocol. BMC Res Notes.

[19] Powell RA, Single HM (1996). Focus groups. International journal for quality in health care : journal of the International Society for Quality in Health Care / ISQua.

[20] Palinkas LA, Horwitz SM, Green CA, Wisdom JP, Duan N, Hoagwood K (2015). Purposeful sampling for qualitative data collection and analysis in mixed method implementation research. Admin Pol Ment Health.

[21] Mayring P. Qualitative Inhaltsanalyse. In: Flick U, Ev K, Reinbek SI, editors. *Qualitative Forschung Ein Handbuch*. 5th ed. Reinbek: Rowohlt Taschenbuch; 2007.

[22] Candy B, Jones L, Drake R, Leurent B, King M. Interventions for supporting informal caregivers of patients in the terminal phase of a disease. Cochrane Database Syst Rev. 2011;(6):CD007617. 10.1002/14651858.CD007617.pub2 PMC13247825 21678368

[23] Grande GE, Farquhar MC, Barclay SI, Todd CJ (2004). Valued aspects of primary palliative care: content analysis of bereaved carers’ descriptions. The British journal of general practice : the journal of the Royal College of General Practitioners.

[24] Krug K, Bölter R, Ballhausen RA, Engeser P, Peters-Klimm F. Burden experienced by family caregivers of patients at the end of life: what do general practice teams offer? Gesundheitswesen (Bundesverband der Ärzte des Öffentlichen Gesundheitsdienstes (Germany)). 2016;78(S 01):e128–34. 10.1055/s-0042-111206 27441824

[25] Slort W, Schweitzer BP, Blankenstein AH, Abarshi EA, Riphagen II, Echteld MA, Aaronson NK, van der Horst H, Deliens L (2011). Perceived barriers and facilitators for general practitioner-patient communication in palliative care: a systematic review. Palliat Med.

[26] Guerriere D, Husain A, Zagorski B, Marshall D, Seow H, Brazil K, Kennedy J, Burns S, Brooks H, Coyte PC (2016). Predictors of caregiver burden across the home-based palliative care trajectory in Ontario, Canada. Health Soc Care Community.

[27] Thoonsen B, Vissers K, Verhagen S, Prins J, Bor H, van Weel C, Groot M, Engels Y (2015). Training general practitioners in early identification and anticipatory palliative care planning: a randomized controlled trial. BMC Fam Pract.

[28] Carduff E, Finucane A, Kendall M, Jarvis A, Harrison N, Greenacre J, Murray SA (2014). Understanding the barriers to identifying carers of people with advanced illness in primary care: triangulating three data sources. BMC Fam Pract.

[29] Kristjanson LJ, Aoun S (2004). Palliative care for families: remembering the hidden patients. Can J Psychiatry.

[30] Steinhauser KE, Clipp EC, McNeilly M, Christakis NA, McIntyre LM, Tulsky JA (2000). In search of a good death: observations of patients, families, and providers. Ann Intern Med.

[31] Escobar Pinzon LC, Claus M, Zepf KI, Letzel S, Fischbeck S, Weber M (2011). Preference for place of death in Germany. J Palliat Med.

[32] Ventura AD, Burney S, Brooker J, Fletcher J, Ricciardelli L (2014). Home-based palliative care: a systematic literature review of the self-reported unmet needs of patients and carers. Palliat Med.

[33] Thomas K (2003). Caring for the dying at home.

[34] Hetzel C, Baumann R, Diekmann J, Frobose I. Description of a multidimensional health program for informal caregivers. Gesundheitswesen (Bundesverband der Arzte des Offentlichen Gesundheitsdienstes (Germany)). 2016;2018;80:S51-S6. 10.1055/s-0042-112814 27756085

[35] Schubert I, Heymans L, Fessler J (2010). General practitioners’ guideline for palliative care. A survey of guideline acceptance in quality circles of primary medical care. Medizinische Klinik (Munich, Germany : 1983).

[36] Behmann M, Junger S, Radbruch L, Schneider N (2012). Public health actions to improve palliative care in Germany: results of a three-round Delphi study. Health Policy.

[37] Neergaard MA, Olesen F, Jensen AB, Sondergaard J (2010). Shared care in basic level palliative home care: organizational and interpersonal challenges. J Palliat Med.

[38] Baile WF, Palmer JL, Bruera E, Parker PA (2011). Assessment of palliative care cancer patients’ most important concerns. Support Care Cancer.

[39] Michiels E, Deschepper R, Van Der Kelen G, Bernheim JL, Mortier F, Vander Stichele R, Deliens L (2007). The role of general practitioners in continuity of care at the end of life: a qualitative study of terminally ill patients and their next of kin. Palliat Med.

[40] Krishnasamy M, Wells M, Wilkie E (2007). Patients and carer experiences of care provision after a diagnosis of lung cancer in Scotland. Support Care Cancer.

